# Identification of Aged Polypropylene with Machine Learning and Near–Infrared Spectroscopy for Improved Recycling

**DOI:** 10.3390/polym17050700

**Published:** 2025-03-06

**Authors:** Keyu Zhu, Delong Wu, Songwei Yang, Changlin Cao, Weiming Zhou, Qingrong Qian, Qinghua Chen

**Affiliations:** 1 College of Environmental and Resource Sciences, College of Carbon Neutral Modern Industry, Fujian Normal University, Fuzhou 350007, China; qbx20210115@yjs.fjnu.edu.cn (K.Z.); qsz20221574@student.fjnu.edu.cn (D.W.); yangsongwei@fjnu.edu.cn (S.Y.); qrqian@fjnu.edu.cn (Q.Q.); 2 Engineering Research Center of Polymer Green Recycling of Ministry of Education, Fujian Normal University, Fuzhou 350007, China; 3 Fujian Key Laboratory of Pollution Control & Resource Reuse, Fuzhou 350007, China

**Keywords:** machine learning, classification, aging, near–infrared spectroscopy, polypropylene

## Abstract

The traditional plastic sorting process primarily relies on manual operations, which are inefficient, pose safety risks, and result in suboptimal separation efficiency for mixed waste plastics. Near–infrared (NIR) spectroscopy, with its rapid and non–destructive analytical capabilities, presents a promising alternative. However, the analysis of NIR spectra is often complicated by overlapping peaks and complex data patterns, limiting its direct applicability. This study establishes a comprehensive machine learning–based NIR spectroscopy model to distinguish polypropylene (PP) at different aging stages. A dataset of NIR spectra was collected from PP samples subjected to seven simulated aging stages, followed by the construction of a classification model to analyze these spectral variations. The aging of PP was confirmed using Fourier–transform infrared spectroscopy (FTIR). Mechanical property analysis, including tensile strength and elongation at break, revealed a gradual decline with prolonged aging. After 40 days of accelerated aging, the elongation at the break of PP dropped to approximately 30%, retaining only about one–sixth of its original mechanical performance. Furthermore, various spectral preprocessing methods were evaluated to identify the most effective technique. The combination of the second derivative method with a linear –SVC achieved a classification accuracy of 99% and a precision of 100%. This study demonstrates the feasibility of the accurate identification of PP at different aging stages, thereby enhancing the quality and efficiency of recycled plastics and promoting automated, precise, and sustainable recycling processes.

## 1. Introduction

Polypropylene (PP) is a semi–crystalline thermoplastic polymer composed of propylene monomers, which is widely utilized in the pharmaceutical, construction, toy, and chemical industries due to its unique physicochemical properties [1]. These properties include a high melting point, low density, and exceptional resistance to chemical solvents and fatigue [2]. Such characteristics stem from its isotactic molecular structure and degree of crystallinity (typically 30–50%), which directly influence its mechanical strength and thermal stability [3]. However, despite these favorable characteristics, PP presents challenges in recycling. As a dominant component of plastic waste, PP accounts for approximately 21% of global recycled plastics, yet its recycling efficiency is hindered by intrinsic material heterogeneity [4].

The aging of PP, induced by environmental stressors such as UV radiation, thermal oxidation, and mechanical wear, further complicates its recyclability and overall lifecycle [5]. Aging triggers chain scission and oxidation reactions, leading to an increased carbonyl index and crystallinity alterations [6]. These structural degradations manifest as reduced tensile strength, embrittlement, and discoloration, collectively termed as “property decay” without effective stabilization measures [7]. Critically, aged PP exhibits distinct physicochemical signatures (e.g., altered melt flow index and increased surface polarity) compared to virgin PP, which significantly impacts its processability and performance in recycled applications and poses challenges for mechanical reprocessing but are often overlooked in conventional recycling workflows. In addition, conventional recycling systems still lack efficient sorting technologies to distinguish these compositional differences, resulting in downcycled products with compromised performance. As a result, advanced sorting technologies capable of discriminating PP are urgently needed. This challenge is not merely technical but also fundamental for achieving circular economy goals, as mismatched PP blends degrade the recyclate quality and limit reuse in high–value applications [8]. The limitations of manual sorting—inefficiency, health risks, and inability to differentiate PP subtypes or aging stages—have driven the adoption of spectroscopic techniques, among which NIR spectroscopy emerges as a gold standard for industrial–scale polymer characterization [9,10,11]. Unlike Raman spectroscopy, which is susceptible to fluorescence interference or mid–infrared (MIR) methods, which require physical contact, NIR enables the non–destructive rapid analysis of bulk materials through conveyor–based systems, while capturing molecular fingerprints critical to aging assessment, such as C–H overtone vibrations and carbonyl group formation [12,13,14]. These spectral features correlate with PP’s structural degradation mechanisms, including chain scission and oxidation, and conventional chemometric tools (e.g., PCA) often fail to resolve them due to overlapping absorbance bands and additive interference [15].

Machine learning (ML) addresses this complexity by automating feature extraction from high–dimensional NIR data: convolutional neural networks (CNNs) spatially map localized degradation patterns, while attention mechanisms prioritize wavelength regions encoding aging gradients [16,17]. Though ML has demonstrated > 90% accuracy in plastic–type classification using Raman/LDIR spectroscopy [15,18], these successes remain confined to controlled laboratory conditions, neglecting real–world challenges such as surface contaminant noise and continuous aging transitions in recycled PP streams [17]. Moreover, the variability in waste PP compositions further complicates the generalization of these methods, limiting their practical application in industrial–scale recycling.

Crucially, despite evidence that even a 5% change in crystallinity in PP can have a significant effect on the mechanical properties of the recyclates [19], there is still a research gap in terms of correlating NIR spectroscopy with time–aging mechanisms. Our work aptly fills this gap, and the aim of this study is to explore the feasibility of using the ML algorithm to distinguish the NIR spectra of PP at different aging stages. The PP material was produced through a molding process and subjected to artificially accelerated UV aging. The spectral data were thoroughly analyzed, including feature extraction, model selection, and evaluation. Aging effects were further analyzed using morphological assessment and related characterization techniques to elucidate the degradation process of PP. Based on these findings, a set of classification and recycling strategies were proposed. ML algorithms were then applied to the subtle spectral changes associated with PP aging to identify and classify PP with different degrees of degradation. This approach is expected to improve the assessment of material degradation, enhance the quality of recycled materials, and provide new methods for efficient and environmentally sustainable plastics recycling, thus contributing to the development of precision recycling in line with the requirements of the circular economy.

## 2. Materials and Methods

### 2.1. Preparation of PP Samples

The PP used in this experiment was sourced from Jinxi Petrochemical (grade K1102). The PP material was processed through injection and extrusion using an MJ55 injection molding machine from Ningbo Zhenxiong Machinery Co., Ltd., Ningbo, China. The extrusion process was carried out at temperatures of 220 °C, 215 °C, and 210 °C across different temperature zones to fabricate standard PP sample strips.

### 2.2. Aging of PP Samples

Prior to the aging test, both the PP sample strips and experimental vessels were cleaned using ultrapure water to eliminate any release agents from the injection molding and extrusion processes, ensuring accurate composition analysis.

Accelerated aging experiments were performed using the LUV–II type UV accelerated aging tester, which simulates the effects of light and water exposure on the PP samples. The UV light wavelength was set at 313 nm, and the samples were exposed to a temperature of 70 °C. The aging cycle consisted of a 12–h irradiation period followed by water replenishment and the repeated cycle, totaling 1200 h of exposure. This method simulates the degradation of PP under environmental conditions induced by solar UV light. Samples were first collected at intervals of 2 days, followed by every 10 days, after which they were dried at room temperature and subjected to further analysis.

### 2.3. NIR Spectral Dataset and Preprocessing

NIR spectra were obtained using a BASF Trinamix NIR spectrometer, covering a spectral range of approximately 1400 nm to 2500 nm. The scan interval was set to 8 nm per time, resulting in a total of 353 acquisitions. Diffuse reflected light was analyzed to collect rapid non–destructive spectrograms. Upon activation, the device was paired with a mobile application for setup, and the raw signals underwent calibration to ensure the spectra represented relative reflectance [20]. The formula used for calibration is as follows:

(1) $$S=\frac{S_{0}-B}{W-B}$$

where $S_{0}$ is the original reflection signals, B is the reference spectrum of the dark current, and W is the reference spectrum of the light source.

To investigate aging effects and performance differences in PP, NIR spectral data for PP samples aged at 2, 10, 20, 30, 40, and 50 days were collected. The obtained spectral information at different aging stages is summarized in Table 1.

Additionally, the second–order derivative method was applied as a spectral preprocessing technique to enhance the analysis of spectral variations across different aging stages of PP. This method effectively reduces the influence of stray light, noise, and baseline drift. The principle is described by the following equation [21]:

(2) $$f^{\prime \prime }\left(x\right)={\left.\left(\frac{d}{dx}\left(\frac{df}{dx}\right)\right)\right|}_{x}$$

where ${\left.\left(\frac{d}{dx}\left(\frac{df}{dx}\right)\right)\right|}_{x}$ indicates that one should first compute the first derivative of $f\left(x\right)$ then differentiate the result once more and finally evaluate the outcome at the point$\left(x\right)$.

### 2.4. Linear –SVC Model Architecture and Training

Linear –Support Vector Classification (SVC) is a widely used ML technique, and linear –SVC applies a linear classification approach in multi–class settings using Support Vector Machines (SVMs). The core principle involves separating data using a linear kernel function as the decision boundary. Linear –SVC offers greater flexibility in selecting penalty and loss functions, demonstrating excellent generalization capabilities, particularly for handling high–dimensional data [22]. The decision function for linear SVC classification is defined as follows [23]:

(3) $$f\left(x\right)=\omega \cdot x+b$$

(4) $$\omega =\sum_{i=1}^{n}a_{i}y_{i}x_{i}$$

(5) $$b=y_{k}-\omega \cdot x_{k}$$

where x represents the feature vector of the input data, y represents the labels of the input data points, ω is the weight vector, b is the bias term, k is any support vector, and $a_{i}$ is the Lagrange multiplier.

The NIR spectral dataset was split into training and testing sets, with the training set used to train the algorithm and the test set for model evaluation [24]. The model used a regularization parameter of 1.0, a linear kernel function, and a hinge loss function, with all other parameters set to their default values. The data underwent preprocessing, which included normalization and error removal using the Hampel filter. Classification results were obtained after 100 iterations. The analysis was conducted using a standalone Python 3.7 script on a server equipped with an Intel Core i7–12700F CPU (12 cores @ 2.10 GHz).

Additionally, confusion matrix and Receiver Operating Characteristic (ROC) curves were utilized to analyze classification results and model performance. Confusion matrices were used to visualize both misclassification and correct classification results, while ROC curves allowed for an evaluation of the model’s ability to distinguish samples at various thresholds [25].

### 2.5. Sample Characterization

Chemical analysis of the samples was performed using a Fourier transform infrared spectrometer (FTIR) (Nicolet 5700, Thermo Fisher Scientific, Waltham, MA, USA, 81 Wyman Street, Waltham, MA, 02454). The samples were prepared by pressing into thin films to ensure uniformity and consistency. The FTIR spectra were acquired in the range of 4000 to 400 cm^−1^ with a resolution of 4 cm^−1^, and an average of 32 scans were taken for each sample to enhance the signal–to–noise ratio.

In accordance with GB/T 1040.2–2006 standards [26], the mechanical properties of the PP specimens at various aging stages were investigated using a microcomputer–controlled electronic universal tensile testing machine ((CMT4104, Nss Laboratory Equipment Co., Ltd., Shenzhen, China). The tensile speed was precisely set to 50 mm/min to ensure consistent testing conditions. The samples were carefully mounted with a sample spacing of 50 mm, a sample width of 10 mm, and a thickness of 4 mm. Each specimen was clamped securely to prevent slippage during the test. The tensile testing was conducted at ambient temperature (23 °C ± 2 °C) and relative humidity (50% ± 5%) to minimize environmental variability.

## 3. Results and Discussion

### 3.1. Characterization of PP at Different Aging Stages

**Figure 1:**
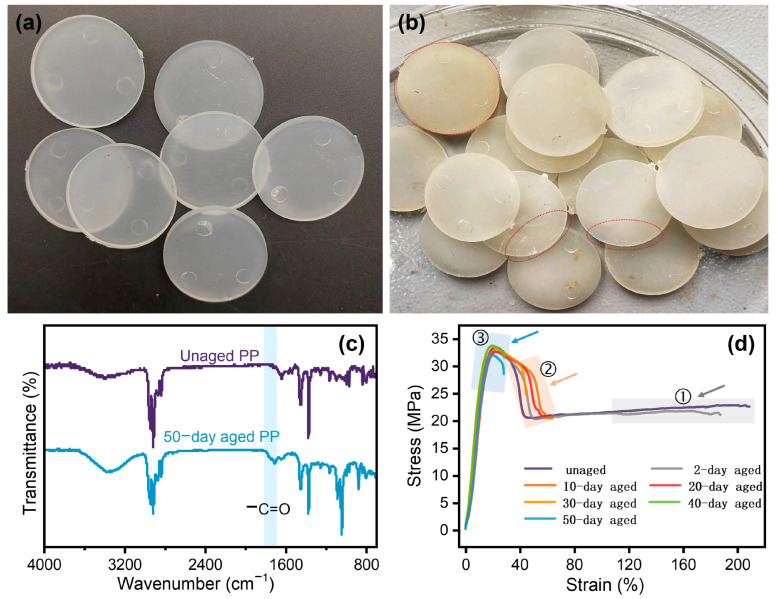
Digital photograph of (**a**) unaged PP and (**b**) 50-day aged PP. (**c**) FTIR spectra of unaged PP and 50-day aged PP. (**d**) Stress−strain curves of unaged PP and aged PP.

Figure 1 shows digital photographs of PP before and after aging. Unaged PP has high transparency, good gloss, and a smooth rounded surface (Figure 1a). In contrast, aged PP shows a significant reduction in gloss, along with yellowing, chalking, and localized cracking (Figure 1b). These changes are indicative of the surface degradation of PP.

FTIR analysis was performed to investigate the chemical changes. Figure 1c shows the characteristic absorption peaks of –CH_3_ bonds at around 1300 cm^−1^ (stretching vibration) and 2900 cm^−1^ (bending vibration), which are present in both unaged and aged PP. This indicates that the basic structural units of PP remain largely unchanged after aging. However, a significant difference is observed in the absorption peak at 1750 cm^−1^ between unaged PP and 50-day aged PP, indicating the presence of carbonyl (C=O) bonds in the aged sample. The observed changes in the appearance and chemical characteristics of aged PP can be attributed to surface oxidation under UV irradiation. This leads to the formation of oxygen–containing functional groups, causing color changes, increased surface roughness, and the chalking and cracking phenomena. The FTIR results indicate that while the basic structural units of PP remain largely unchanged, the presence of carbonyl bonds (C=O) at 1750 cm^−1^ in the aged sample confirms the formation of oxidation products. Overall, these results highlight the significant impact of UV–induced oxidation on the chemical and morphological properties of PP.

The stress––strain curves of PP at various aging stages are shown in Figure 1d. The tensile strength of PP decreases progressively with aging, especially after 50 days. Similarly, the elongation at break decreases over time. The change process can be summarized into three distinct stages:

Stage ① (Aged for 2 days): The elongation at the break is above 180%, indicating that the properties of the PP sample after two days of aging are almost equivalent to those of the unaged sample;

Stage ② (Aged for 10–30 days): The elongation at break drops to around 70%, marking a significant change in properties. The performance of PP remains relatively stable from 10 to 30 days of aging;

Stage ③ (Aged for 40–50 days): The elongation at the break further decreases to around 30%, indicating a substantial reduction in toughness.

The progressive reduction in tensile strength and elongation at the break with aging suggests significant degradation of the polymer chains. This degradation can be attributed to the rupture of C–H bonds under UV light and elevated temperatures, leading to the formation of free radicals and subsequent oxidation products. The three distinct stages of degradation identified in the stress––strain curves help inform subsequent sorting and recycling strategies for PP waste. In Stage 1, the properties of PP remain within a relatively stable range and can therefore be recycled directly. In Stage 2, PP still has value, but physical recycling may be necessary to improve its properties. In Stage 3, PP shows reduced properties, and chemical recycling via cracking may be more economically feasible than physical recycling. This classification strategy, based on the aging gradient, can improve recycling efficiency and extend the life cycle of plastic products [27].

### 3.2. NIR Spectral of PP at Different Aging Stages

**Figure 2:**
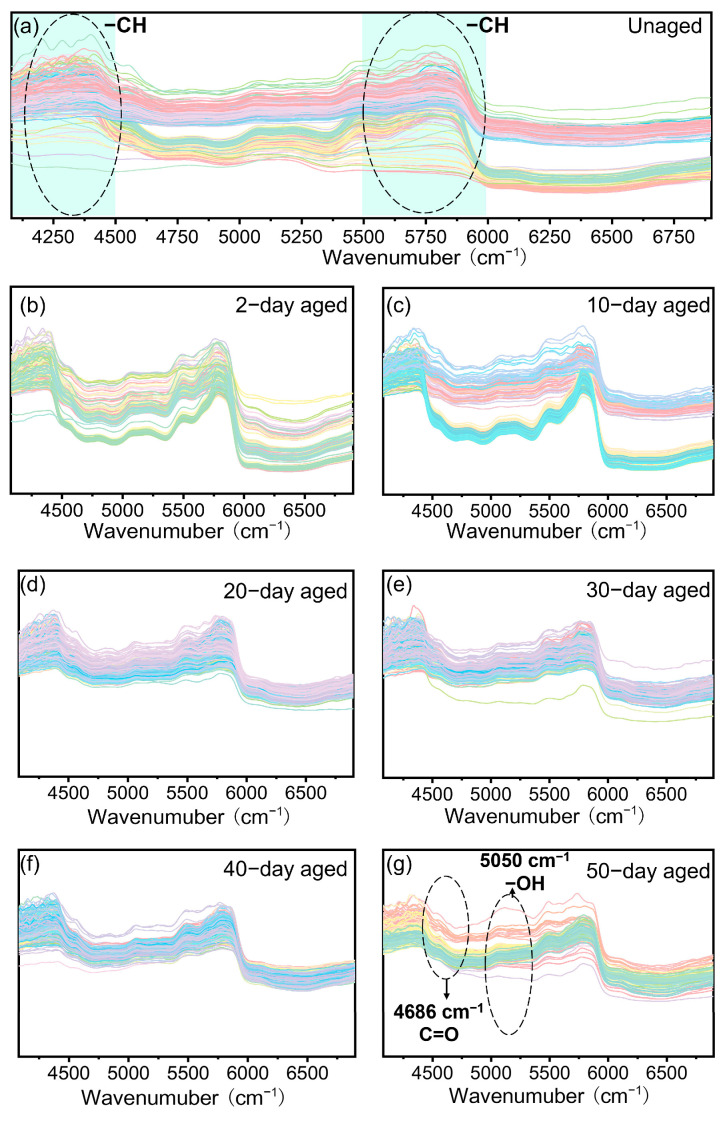
NIR spectra of PP at different aging stages. (**a**) Unaged, (**b**) 2-day aged, (**c**) 10-day aged, (**d**) 20-day aged, (**e**) 30-day aged, (**f**) 40-day aged, and (**g**) 50-day aged.

Figure 2 presents the NIR spectral information of PP at various aging stages, offering valuable insights into the molecular changes occurring throughout the aging process. Initially, the NIR spectra of unaged PP (Figure 2a) display closely aligned spectral profiles, with characteristic absorption peaks primarily between 4000 and 4500 cm^−1^ and between 5500 and 6000 cm^−1^. These absorption peaks are attributed to the monomer structure of PP and the vibrations of –CH, –CH_2_, and –CH_3_ bonds [28].

As PP ages, the overall shape of the NIR spectral curves remains consistent, indicating that the internal structure of PP does not undergo significantly change under aging conditions (Figure 2b–g). This finding is consistent with the FTIR results, which also suggest that the basic structural units of PP remain largely unchanged. However, subtle variations in the NIR spectra indicate modifications in the molecular structure and characteristics of PP. For instance, Figure 2b shows no significant changes in the NIR spectral curve, implying minimal impact on the molecular structure after initial exposure to aging conditions.

In contrast, Figure 2c reveals additional absorption peaks between 5000 and 5500 cm^−1^, along with the characteristic peaks at 4000–4500 cm^−1^ and 5500–6000 cm^−1^. This suggests the formation of new functional groups or structural changes, likely due to the oxidation of PP under UV irradiation. Figure 2d shows closely stacked spectral curves with more intense absorption at 4000–4500 cm^−1^, indicating enhanced structural changes or the formation of new bonds. Figure 2e demonstrates a narrowing of the absorption peaks, which is indicative of the breaking of PP molecular chains and a decrease in molecular weight. Figure 2f shows a shift in the absorption peaks between 5000 and 5500 cm^−1^, indicating a change in the crystallinity of PP. This suggests alterations in the packing and alignment of polymer chains, consistent with the mechanical test results, which show a decrease in tensile strength and elongation at break. Figure 2g displays a more pronounced change in the characteristic absorption peaks, with the appearance of new peaks and changes in the strength of existing signals. This indicates significant modifications in the molecular structure and functional groups of PP [29,30].

These changes in the NIR spectra, combined with the findings from FTIR and mechanical tests, provide a comprehensive understanding of the effects of prolonged aging on PP. While the overall shape of the NIR spectral curves suggests that the internal structure of PP remains largely unchanged, the subtle shifts and new peaks point to significant modifications in molecular structure and properties. The appearance of new functional groups and changes in crystallinity are consistent with the oxidation and chain scission processes observed during aging. These results highlight the importance of considering both structural and chemical changes when assessing the degradation of aged PP. The combined analysis of NIR, FTIR, and mechanical test results demonstrates that prolonged aging leads to significant modifications in the molecular structure and properties of PP, ultimately affecting its mechanical performance and recyclability.

### 3.3. Data Preprocessing

Spectral preprocessing plays a vital role in enhancing the quality of spectral data, thereby improving the accuracy and reliability of subsequent analyses while minimizing errors [31]. This study focuses on five spectral preprocessing methods: first–order derivative, second–order derivative, standard normal variate (SNV), multiple scatter correction (MSC), and moving average, which aim to mitigate various issues that could hinder model training, such as variations in illumination, noise, light scattering effects, and subtle peak characteristics.

**Figure 3:**
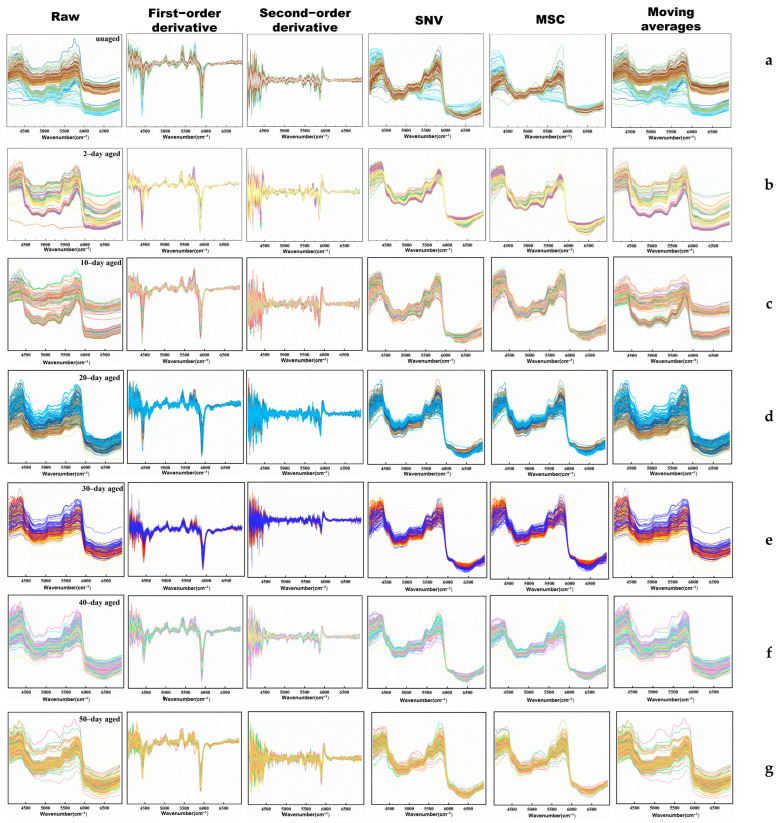
Comparison of different spectral pretreatment methods for different aging stages of PP. (**a**) Unaged (**b**) 2-day (**c**) 10-day (**d**) 20-day (**e**) 30-day (**f**) 40-day and (**g**) 50-day.

Figure 3 shows a comparison of the NIR spectral profiles after preprocessing, applied to PP spectra with different aging times (2-day, 10-day, 20-day, 30-day, 40-day, and 50-day). The preprocessing methods aim to address common issues in spectral data, such as noise, scattering effects, and baseline variations, thus enhancing the quality of the data for subsequent analysis.

The first column of Figure 3a–g demonstrates the effects of first–order derivative preprocessing. This method accentuates local maxima and minima features, enhancing the ability to detect subtle changes in the spectra. However, it also has the potential to amplify noise and obscure critical spectral details. As shown, the first–order derivative effectively enhances local features, but this comes at the risk of introducing noise, particularly in the spectra of younger samples (e.g., 2 days and 10 days).

The second column of Figure 3 highlights the effects of second–order derivative preprocessing. This method is particularly effective in addressing the issue of peak overlap and accentuating subtle variations in highly similar curves [32]. It is evident that the second–order derivative method successfully resolves overlapping peaks and enhances the visibility of minor spectral changes, making it highly effective for analyzing complex spectra with closely spaced features. This preprocessing technique is especially beneficial for distinguishing between the spectra of PP samples with different aging stages.

The third column of Figure 3 shows the results of the SNV preprocessing method. This technique reduces differences in the physical properties of spectral data by subtracting the mean and dividing by the standard deviation [33]. In this study, the standard deviation between data points is small, resulting in a relatively modest processing effect compared to the original data. While SNV helps normalize the spectra, it does not significantly alter the overall trends, thereby making it a useful preprocessing step for baseline correction.

The fourth column of Figure 3 illustrates the effects of the MSC method. This technique effectively eliminates spectral variations caused by scatter and enhances the correlation between spectra and data [33]. However, MSC requires a substantial amount of data and ideal spectra for optimal results. In this study, MSC preprocessing helps to reduce the impact of scattering effects, thus improving the consistency of the spectra. This preprocessing method is particularly beneficial for aligning spectra and enhancing the signal–to–noise ratio.

The fifth column of Figure 3 shows the results of the moving average preprocessing method. This technique smooths the data series to reduce random fluctuations [34]. As demonstrated, the moving average preprocessing method has a minimal impact on the overall spectral shape, preserving the original trends while reducing noise. This method is particularly useful for smoothing short–term fluctuations and highlighting longer–term trends in the spectra.

In conclusion, the preprocessing methods significantly improve the quality of the NIR spectra, protecting them from environmental noise and distortions. The first–order and second–order derivative methods are particularly effective in enhancing local features and resolving peak overlap, respectively. The SNV, MSC, and moving average methods help to normalize and smooth the spectra, preserving the original information without significantly altering the overall trends. These preprocessing techniques facilitate the construction of machine learning models by reducing noise and improving the consistency of the spectral data. Therefore, the subsequent experiments primarily compare the first– and second–order derivative preprocessing methods, owing to their ability to highlight subtle changes and resolve complex spectral features.

### 3.4. Machine Learning Outcome

A linear–SVC classification algorithm was employed to classify PP at different aging times. The performance of the model was evaluated using two spectral preprocessing methods: first–order derivative and second–order derivative. Table 2 shows the classification results of the model using the first–order derivative preprocessing method. The model achieved an overall accuracy of 98%.

Using this preprocessing method, the trained linear –SVC model achieved 100% detection rates for unaged PP, 2-day aged, 10-day aged, and 40-day aged PP. However, the classification accuracy dropped for 20-day, 30-day, and 50-day aged PP. This inconsistency can be attributed to the lack of significant differences in the NIR spectra for PP aged at these stages, which resulted in misclassification. These results are reflected in the precision, recall, and F1–score for this model, as shown in Table 3, and the relevant formulas are set out in the Supplementary Material (Table S1).

When using the second–order derivative preprocessing, the model’s classification accuracy improved to 99%. The performance metrics for the second–order derivative method are detailed in Table 4.

The precision, recall, and F1 scores of the model using the second–order derivative preprocessing method to classify PP aging are shown in Table 5. The precision reaches 100% for most aging stages, except for 30-day and 50-day aged PP. Similarly, the recall reached 100% for most stages, except for 10-day, 20-day, and 40-day aged PP. This suggests that misclassification and omission are more likely to occur in these two categories of 30-day and 50-day aged PP, which leads to a decrease in recall of the other categories. This phenomenon may be attributed to the limited distinction in PP performance between the 30-day and 50-day aged PP. Despite this, the overall performance of the second–order derivative preprocessing method outperforms that of the first–order derivative method. This supports the suitability of the second–order derivative approach for preprocessing PP samples with varying levels of aging prior to machine learning model training.

### 3.5. Visualization Results of Model Training with Different Preprocessing Methods

**Figure 4:**
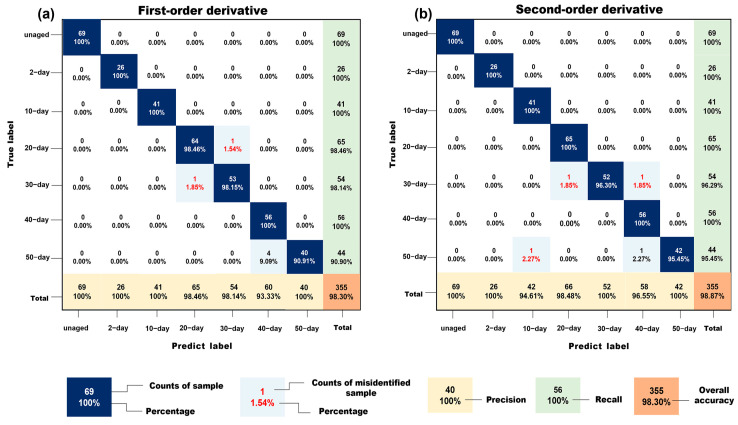
Confusion matrix diagram of first–order derivative and second–order derivative preprocessing methods.

The confusion matrix provides a detailed visualization of the classification results of different preprocessing methods, helping to assess model performance and identify specific areas of misclassification. Figure 4 shows the confusion matrix for models trained using first–order and second–order derivatives as preprocessing methods. The colors of the matrix cells represent different evaluation metrics, while the values and percentages displayed within the cells represent the number of samples in the test set and the associated precision, respectively.

Figure 4a presents the confusion matrix for the model using first–order derivative preprocessing. This model achieved a precision rate of 100% for unaged PP, 2-day aged, 10-day aged, and 40-day aged PP. However, certain misclassifications were observed for 20-day, 30-day, and 50-day aged PP. Specifically, one sample from the 20-day aged group was incorrectly classified as 30-day aged PP, one sample from the 30-day aged group was misclassified as 20-day aged, and four samples from the 50-day aged group were misidentified as 30-day aged PP. Additionally, one sample from the 50-day aged group was classified as 20-day aged PP, and one sample from the 30-day aged group was mistaken for 50-day aged PP. Furthermore, four samples from the 50-day aged group were misclassified as 40-day aged PP. These misclassifications primarily occurred between the 20, 30, and 40-day aging categories. This resulted in reduced precision and recall for these periods, thereby negatively impacting the overall classification accuracy. The confusion matrix analysis highlights that the first–order derivative method faces challenges in distinguishing between the spectra of PP aged for 20, 30, and 50 days, which have subtle differences in their NIR spectra.

Figure 4b illustrates the confusion matrix for the model using second–order derivative preprocessing. This model achieved 100% accuracy for all aging periods except for 20 and 50 days, outperforming the first–order derivative model. For the 30-day aged PP group, two samples were misclassified—one as 20-day aged PP and the other as 40-day aged PP. Similarly, two samples from the 50-day aged group were misclassified—one as 40-day aged PP and the other as 10-day aged PP. Misclassifications in this model were primarily between the 10-day, 20-day, and 40-day aging categories. Despite these errors, the overall classification accuracy was higher than that of the first–order derivative model. The confusion matrix analysis shows that within the test set, six data points were misclassified using the first–order derivative method, while only four were misclassified using the second–order derivative method. This indicates that the model utilizing second–order derivatives as a preprocessing method achieves superior overall classification accuracy, as further corroborated by the data in Table 4.

The confusion matrix analysis provides valuable insights into the performance of the models using different preprocessing methods. The first–order derivative method, while effective for distinguishing between unaged and lightly aged PP, struggles with spectra of PP aged for 20, 30, and 50 days. This is likely due to the subtle differences in NIR spectra for these aging stages, which can lead to misclassification. In contrast, the second–order derivative method demonstrates superior performance, achieving higher overall accuracy and fewer misclassifications. This method is particularly effective in resolving overlapping peaks and subtle variations in the spectra, making it well–suited for distinguishing between highly similar curves. The results suggest that the second–order derivative preprocessing method is more effective at enhancing the distinctiveness of NIR spectra for different aging stages of PP. This preprocessing technique helps to mitigate the challenges posed by subtle spectral differences, thereby improving the accuracy and reliability of the classification model.

**Figure 5:**
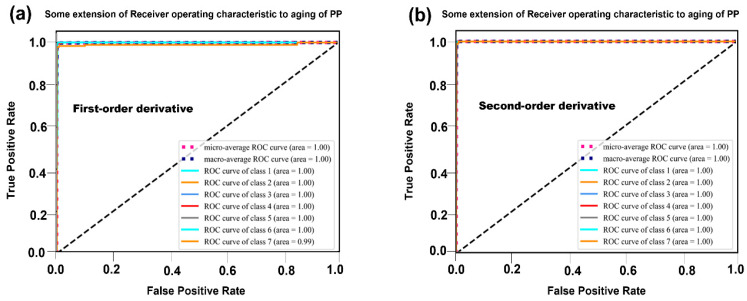
ROC curve of different preprocessing methods. (**a**) First–order derivative method and (**b**) second–order derivative method.

Figure 5 compares the ROC curves for models using first–order and second–order derivative preprocessing methods, providing insights into their classification performance. Both models exhibit exceptionally high Area Under the Curve (AUC) values, suggesting that both preprocessing methods lead to highly effective classifiers. Even with adjustments to the decision thresholds, the performance of both models remains robust and well–calibrated.

In Figure 5a, the ROC curve analysis for the first–order derivative model shows that all categories, except for the 50-day aged PP, achieved a perfect AUC value of 1. The 50-day aged PP category exhibited an AUC value of 0.99. This minor deviation from a perfect score indicates that the first–order derivative method is highly effective in distinguishing between most aging stages of PP. However, the slight drop in AUC for the 50-day aged PP suggests that this category may have subtle spectral differences that are more challenging to resolve using this preprocessing method.

Figure 5b presents the ROC curve analysis for the second–order derivative model, which shows that all categories achieved a perfect AUC value of 1. This result demonstrates that the second–order derivative method consistently outperforms the first–order derivative method in classifying PP samples across all aging stages. The robust performance of the second–order derivative model, even for the most challenging category (50-day aged PP), highlights its superior ability to capture subtle spectral variations and enhance the distinctiveness of the NIR spectra.

The ROC curve analysis provides a comprehensive evaluation of the classification performance of the models using different preprocessing methods. The exceptionally high AUC values observed for both models indicate that both preprocessing techniques are highly effective in distinguishing between different aging stages of PP. However, the second–order derivative method demonstrates a slight yet statistically significant advantage as it achieves perfect AUC values across all categories, including the more challenging 50-day aged PP.

This superior performance can be attributed to the second–order derivative’s ability to resolve overlapping peaks and highlight subtle variations in the spectra, which are critical for accurately classifying highly similar curves. As shown in the results of Table 5, this conclusion is further substantiated, with the second–order derivative method consistently outperforming the first–order derivative method in terms of precision, recall, and F1–score.

In summary, the ROC curve analysis strongly supports the notion that the second–order derivative preprocessing method is more effective in enhancing the distinctiveness of NIR spectra for different aging stages of PP. This method offers a more robust and reliable classification performance, positioning it as a preferred choice for preprocessing PP samples prior to machine learning model training.

## 4. Conclusions

While traditional manual plastic classification suffers from inherent limitations in efficiency and accuracy in aging stage differentiation, this study introduces a groundbreaking algorithmic model for intelligent plastic classification through NIR spectral characterization. The model systematically optimizes the NIR spectral preprocessing workflow, where the second–order derivative transform eliminates baseline drift interference while enhancing age–sensitive spectral features, enabling the linear SVC model to achieve an unprecedented 99% recognition accuracy and demonstrating remarkable generalization across different sample variations.

Our approach bridges the existing technological gap by combining a second–order derivative preprocessing method with spectral feature selection, thereby overcoming the reliance on traditional full–spectrum analysis. This combination explains that the aging carbonyl group and the PP’s own C–H bond are the keys to successful classification, while also validating the underlying classification logic. In addition, the model is both lightweight and robust, with scalability to multi–polymer systems. From a sustainability standpoint, the accuracy of automated sorting could significantly improve recycling purity compared to current optical sorting systems, diverting more and more waste from landfills to closed–loop recycling [35].

Future work will focus on utilizing federated learning to establish cloud–based model updating mechanisms, ensuring sustained performance for the global material stream, as well as investigating fusion architectures for simultaneous aging classification and mechanical property prediction.

## Figures and Tables

**Table 1:** Number of NIR spectral data before and after PP aging.

Aging Time (Days)	Amount of Data (Items)
0	324
2	182
10	234
20	294
30	285
40	266
50	189
Total	1774

**Table 2:** Results of PP at different aging stages trained by first derivative.

Type	Preprocessing	Classification Method	Accuracy (%)
unaged	First–order derivative	Linear–SVC	98
2-day			
10-day			
20-day			
30-day			
40-day			
50-day			

**Table 3:** Precision, recall, and F1–score of first–order derivative method.

Method	Type	Precision (%)	Recall (%)	F1–Score
First derivative method + Linear–SVC	unaged	100	100	1.00
	2-day	100	100	1.00
	10-day	100	100	1.00
	20-day	98	98	0.98
	30-day	98	98	0.98
	40-day	100	93	0.97
	50-day	91	100	0.95

**Table 4:** Results of PP at different aging stages trained by the second–order derivative.

Type	Preprocessing	Classification Method	Accuracy (%)
unaged	Second–order derivative	Linear–SVC	99
2-day			
10-day			
20-day			
30-day			
40-day			
50-day			

**Table 5:** Precision, recall, and F1–score of the second–order derivative method.

Method	Type	Precision (%)	Recall (%)	F1–Score
Second derivative method + Linear–SVC	unaged	100	100	1
	2-day	100	100	1
	10-day	100	98	0.99
	20-day	100	98	0.99
	30-day	96	100	0.98
	40-day	100	97	0.98
	50-day	95	100	0.98

## Data Availability

Data is contained within the article or Supplementary Material. The data presented in this study are available on request from the corresponding author.

## Supplementary Materials

The following supporting information can be downloaded at: https://www.mdpi.com/article/10.3390/polym17050700/s1, Table S1: Calculating formulas on precision, recall, and accuracy.
