# Supplementary material for: Identification of Aged Polypropylene with Machine Learning and Near–Infrared Spectroscopy for Improved Recycling
Source: Polymers (Basel). 2025 Mar 6;17(5):700. doi: 10.3390/polym17050700 (PMC11902415; doi:10.3390/polym17050700)
Supplement: Supplementary file 1 [file polymers-17-00700-s001.zip › polymers-3480016-supplementary.pdf]

Supplementary material

Table S1 Calculating formulas on precision, recall, and accuracy

$$Precision = \frac{TP}{TP + FP}$$	(1)
$$Recall = \frac{TP}{TP + FN}$$	(2)
$$Accuracy = \frac{TP + TN}{TP + TN + FP + FN}$$	(3)

Where TP is true positive, a sample that is actually positive and correctly predicted to be positive, FP is false positive, a sample that is actually negative and incorrectly predicted to be positive, FN is false negative, a sample that is actually positive and incorrectly predicted to be negative, and TN is true negative, a sample that is actually negative and incorrectly predicted to be negative. TN stands for true negative, i.e. a sample that is actually negative and was correctly predicted to be negative.
